# Immunotherapeutic Applications of NK Cells

**DOI:** 10.3390/ph8020250

**Published:** 2015-05-25

**Authors:** Carter T. Davis, David Rizzieri

**Affiliations:** Department of Medicine, Duke University Medical Center, Durham, NC 27710, USA; E-Mail: carter.davis@duke.edu

**Keywords:** Natural Killer cells, hematopoietic stem cell transplant, leukemia, cellular therapy

## Abstract

Natural Killer (NK) cells are lymphoid cells that exhibit an innate response against virus-infected cells. These cells are also capable of mounting an immune response against tumor cells after education through major histocompatibility complex (MHC) class I molecules. NK cell regulation is mediated through IFN-gamma and IL-15, important cytokines which can drive NK cell expansion *in vivo*. Previous studies have shown effective infusion of allogeneic NK cells after lymphodepleting regimens with induction of remission of poor prognosis acute myeloid leukemia (AML). Challenges remain in the expansion of these NK cells once infused and in their education to recognize tumor targets. A principal mechanism of tumor recognition is through KIR mismatch in cells lacking self MHC I molecules. Activating KIRs exist, though their ligands are unknown at this time. Impacting NK cell expansion and education *in vivo* has been challenging, and thus far clinical applications of NK cells have shown promise in helping to maintain remission in humans, though this remission has not been maintained. Future efforts to utilize NK cells clinically are focusing on developing more consistency in successful expansion of NK cell and educating them to recognize their tumor targets. Additional efforts to utilize novel antibody-based therapy to engage NK cells to their tumor targets are also in development.

## 1. Introduction

Natural Killer (NK) cells are cells of lymphoid origin that play key roles eliminating host viral infections as well as the development of tumor cells [1]. NK cells have been observed to eliminate tumor cells both *in vitro* and *in vivo* [2]. Moreover, mouse models that are lacking or have reduced populations of NK cells are more prone to develop tumors such as sarcomas, intestinal neoplasms, pulmonary neoplasms, and lymphomas [3,4,5,6]. These cells are defined in humans by their expression of surface markers of CD56 or CD16 and with the absence of CD3 [7]. Other important markers of NK cells include the natural cytotoxicity receptors (NCRs): NKp30, NKp44, and NKp46, the latter of which is a shared surface marker in humans and mice [8]. NK cells are known to secrete cytokines, such as IFN-γ in response to stimulation by other interleukins, predominantly IL-15 [9]. It is this regulation by cytokines that has formed the basis of some approaches to activating natural killer cells in therapeutic approaches with infusion of allogeneic haploidentical natural killer donor cells to treat cancer [10].

NK cells contain receptors that may activate or inhibit NK cell activity [11]. Receptors characterized include major histocompatibility complex (MHC) class I ligand killer-cell immunoglobulin-like receptors (KIRs) [12]. KIRs can serve as activating receptors or inhibitory receptors [12,13]. Inhibitory KIRs have a transmembrane domain with long cytoplasmic tail. Activating KIRs have shorter cytoplasmic tails and activate NK cells by association with adaptor molecules. A central premise behind effective use of allogeneic natural killer cells is in recognizing mismatches between donor inhibitor KIRs and recipient ligands that may lead to senescence of infused NK cells [1,14]. Other NK cell receptors important receptors involved in signaling include NK group protein 2A (NKG2A) and immunoglobulin-like transcript 2 (ILT-2) [15].

NK cells can target and induce apoptosis through two major pathways [16]. Cellular membranes are disrupted by perforin, and release of granzymes (a family of serine proteases) also serves to disrupt cellular architecture and cause cell death [16]. When NK cells become activated, killing can also occur through CD16 and antibody-dependent cellular cytoxicity (ADCC) on target tumor cells [16]. These cells become opsonized with IgG and subsequently have apoptosis induced through the Fas/Fas-L pathway [17]. Other components of NK cell mediated apoptosis include TNF related apoptosis-inducing ligand or Apo2-lingand (TRAIL/Apo2L). These ligands are able to engage targets that contain death domains. TRAIL specifically has been implicated in controlling tumor cell and metastasis growth [16].

The inherent tumor-killing capacity of NK cells provides a therapeutic modality to treat cancerous cells and especially improve the efficacy of allogeneic stem cell transplantation in achievement of long-term remission of malignancies. Many efforts have been directed at developing an effective transplantation protocol of NK cells and conditioning these cells to be effective in reducing relapse of disease.

## 2. Affecting NK Cell Expansion

In order to utilize NK cells for therapeutic strategies, NK cell lines must be expanded and appropriately educated to their desired targets. NK cells have been expanded from peripheral blood, umbilical cord blood, human embryonic stem cells, induced pluripotent stem cells, and the bone marrow [18]. A variety of protocols that explore methods to expand NK cell lines have been examined. Peripheral blood NK cells have been expanded *ex vivo* with Epstein-Barr Virus transformed B-cell lymphoblastoid cell lines [19]. Also explored with peripheral blood stem cells is expansion using K562 feeder cells transduced with IL-15 or IL-21 and CD137 [20]. Umbilical cord blood has been a rich source of NK cells, which have also been expanded through cell culture and showing promising expansion *ex vivo* as well as the capacity to expand *in vivo* based on mouse models [21,22].

## 3. Education of NK Cells

Educating NK cells to recognize target tumor cells is a challenging yet important component in the effective use of NK cells. Leukemia cells are proposed to use a combination of molecular mimicry of inhibitory signals and other mechanisms of immune suppression to evade the NK cells response. The KIR gene clusters of NK cells lead to two distinct haplotypes, termed A and B. Group A haplotypes have only inhibitory KIRs, whereas group B has both inhibitory and activating KIRs [23,24].

Interaction between HLA Class I antigens and inhibitory KIRs result in tolerance to self. Cells that do not express these HLA Class I antigens are rapidly cleared from circulation by NK cells. It has been observed that when donor NK cells are infused into recipients, those recipients who lack HLA Class I ligand that is cognate for the KIR receptor have increased NK cell tumor lysis *in vitro* [25]. This observation has led to theory that a KIR-mismatched recipient is more likely to have increased NK cell activity against tumor cells than one whose HLA Class I ligand is cognate for the donor NK cells’ KIR inhibitory receptor. This has been demonstrated in T-cell depleted donor lymphocyte infusions that are haplotype mismatched in acute myeloid leukemia, with increase in survival demonstrated [26]. An additional measure to include the outcome of donor NK cell infusions is T-cell depletion with anti-thymocyte globulin.

Unfortunately, ligands for activating KIRs are unknown at this time. This has limited rigorous study of this issue, but there have been important findings regarding activating KIRs to date. Patients who have group B clusters of KIR genes (containing both inhibitory and activating KIRs) have been found to have higher rates of sustained remission and longer survival than those with group A (inhibitor KIRs only) clusters. It has also been observed that recipients whose donors contained activated KIR genes had lower rates of CMV reactivation following HLA-matched related donor transplant. Further study to understand how to utilize these activating KIRs in clinical practice may help improve outcomes associated with NK-cell transfer.

## 4. Clinical Applications of NK Cells

Initial approaches to NK cell therapy involved using IL-2 to stimulate and expand autologous NK cells to treat cancers. While daily low-dose IL-2 had been shown to increase the population of NK cells in up to 20% of patients, *in vitro* studies called into question the cytotoxic potential of these cells. NK cells go through a process known as education to become tolerant to self-MHC molecules, and it was hypothesized that this self-tolerance led to inability to achieve the necessary cytotoxic response of autologous NK cells. Attention was therefore turned to allogeneic transplantation of NK cells to treat high-risk cancers [27].

The first reported trial of successful adoptive transfer and expansion of haploidentical NK cells was by Miller and colleagues at the University of Minnesota in 2005 [10]. In a 43 patient trial, patients with metastatic melanoma, high-risk acute myeloid leukemia (AML), metastatic renal cell carcinoma and one with refractory Hodgkin’s lymphoma were enrolled. Peripheral blood was collected from haploidentical donors and subsequently CD3 depleted and incubated overnight in IL-2. Three different preparative regimens were used (high dose cyclophosphamide + fludarabine, low-dose cyclophosphamide + methylprednisolone, and fludarabine alone). Following infusion of donor NK cells, subjects received IL-2 for two weeks. Of these preparative regimens, only the high-dose cyclophosphamide and fludarabine regimen was sufficiently immunosuppressive to enable expansion of the donor NK cells following transplant. Of the 15 patients who had the high-intensity regimen, eight had at least 1% engraftment of donor NK cells at day 7 or beyond after infusion. Miller and colleagues showed that this regimen induced IL-15 expression, which has previously been shown to drive NK cell differentiation. In contrast, the low-intensity preparative regimen (where NK cells did not engraft and expand) did not induce IL-15 expression. Importantly, in the patients with AML who engrafted, five achieved a complete remission (CR), and four of these patients were KIR-ligand mismatched, supporting this as a strategy to enhance NK cell activity against cancer cells. While initial results were promising, the expansion of NK cells did not prove durable and all patients eventually relapsed.

Use of NK cells to treat other forms of cancer has been performed as well. Results vary for different tumor types. In studies of NK cell infusions in patients with breast and ovarian cancer, expansion of donor NK cells failed. Results were similarly poor for patients with relapsed and refractory Hodgkin’s lymphoma. NK cell therapy has achieved remission in some patients with multiple myeloma. While trials have investigated use of NK cells in a variety of cancers, most focus on hematologic malignancies, and particularly AML.

Clinical trials of haploidentical HSCT protocols with KIR-mismatched donor cells have shown improved results with respect to relapse rates when compared with KIR-matched relative donors. There are some trials, though, that fail to demonstrate this improvement, which has been interpreted as to suggest that factors such as optimization of expansion and education of KIR-mismatched donor cells plays an important role in effective graft versus leukemia effect. Adding to the complexity is that differences in conditioning regimens likely affect the uptake of transplanted donor cells. Being able to improve the uptake and *in vivo* expansion of these NK cells is likely to be important in GVL effect as well as reduction of relapse, as NK cells are among the first donor cells to expand after allogeneic HSCT. Furthermore, higher NK cell counts have been correlated with reduced rates of relapse.

Safety of infusion of NK cell delivery has been shown in patients receiving donor lymphocyte infusions in allogeneic stem cell transplant [28]. In two clinical trials designed to test the safety of T-cell donor lymphocyte infusions enriched with NK cells, the safety and efficacy of NK cell transfer in donor lymphocyte infusions (DLI) was demonstrated. In these studies, 30 patients who had T-cell depleting non-myeloablative allogeneic stem cell transplant (haploidentical in one study and fully matched in the second) received NK-cell enriched donor lymphocyte infusions for high-risk diseases (defined as high risk cytogenetics or those in second or greater remission). Peripheral blood apheresis to collect NK cells, followed by selection with a CD56 antibody was performed and infused into patients in these protocols. In matched donors, a median of 10.6 × 10^6^ NK cells were infused, and in mismatched donors, an average of 9.2 × 10^6^ NK cells were infused. Graft versus host disease was minimal in these patients, and after 1 year of follow-up overall survival was approximately 42% in each group, with surviving patients in remission. NK cell function was measured and increased following NK cell enriched DLI in patients who had not immediately responded post-transplant through this approach. A future phase 3 study to evaluate the efficacy of NK cell enriched DLI is ongoing.

## 5. Future Directions

NK cells are a promising arm of therapy in the treatment of hematologic malignancies and may prove useful in hematopoietic stem cell transplantation. There has been wide variability in responses to infusions of donor NK cells to date, and there are a combination of factors that pose challenges to utilizing these cells as therapy effectively. Among these challenges are identifying an optimal method for expansion *ex vivo*, developing an appropriate conditioning regimen to facilitate engraftment of NK cells, and developing protocols that demonstrate greater consistency of *in vivo* expansion of donated NK cells. Optimizing and standardizing the education of NK cells to their tumor targets may also lead to enhanced tumor killing and improved outcomes. Current chemotherapeutic candidates in this regard are proteasome inhibitors and histone deacetylase compounds.

Miller and colleagues have described bispecific and trispecific antibody derivatives, termed bispecific killer engagers (BiKEs) and trispecific killer engagers (TriKEs) that may help engage NK cells via the CD16 receptor with tumor cells (via CD19 and CD22 receptors) [27]. This may ultimately prove to be a useful platform for enhancing NK cell activity against tumor cells in a variety of settings.

The ideal goal would be to develop a strategy through chemotherapeutic means that activates endogenous NK cells to recognize their tumor cell targets without the need for donor NK cells. The insights over the past decade and progress made in understanding the mechanics of NK cell therapy will likely lead to new cell-based therapeutic strategies in the future.
